# Mental health literacy in adolescents: ability to recognise problems, helpful interventions and outcomes

**DOI:** 10.1186/s13034-017-0176-1

**Published:** 2017-08-15

**Authors:** Udena Ruwindu Attygalle, Hemamali Perera, Bernard Deepal Wanniarachchi Jayamanne

**Affiliations:** 1 Teaching Hospital Karapitiya, Galle, Sri Lanka; 2 Faculty of Medicine, University of Colombo, Colombo, Sri Lanka; 3 National Dengue Control Unit, Ministry of Health, Public Health Complex, Colombo 5, Sri Lanka

**Keywords:** Mental health, Mental health literacy, Adolescents, Help seeking, Referral intentions

## Abstract

**Background:**

Although mental health literacy has been widely studied in adults, there are still relatively few studies on adolescent populations. In Sri Lanka, adolescents account for about one fifth of the population. Current evidence shows that most mental health problems diagnosed in adulthood begin in adolescence. There is also growing evidence that the trajectories of these disorders can be altered through early recognition and intervention. Although, help-seeking for mental health problems is known to be poor in adolescents, mental health literacy improves help-seeking. It is also known that adolescents may act as agents of change regarding mental health in their wider communities. Thus, mental health literacy in adolescents is an important aspect of community mental health initiatives. The objective of this study was to describe aspects of mental health literacy in terms of ability to recognise problems, helpful interventions, helpful referral options and outcomes in a target adolescent population in Sri Lanka. The association between socio economic variables and recognition of mental health problems was also examined.

**Methods:**

This descriptive cross sectional study used a pretested questionnaire on 1002 adolescents aged between 13 and 16, where mental health literacy was assessed using 4 case vignettes. The vignettes represented depression with suicidal ideation, social phobia, psychosis and diabetes, where the last was for comparison.

**Results:**

The response rates for recognition as a mental health problem was 82.2% (n = 824) for the vignette depicting depression, 68.7% (n = 689) for the psychosis vignette and 62.3% (n = 623) for the social phobia vignette. “Talking to the person”, was responded to as helpful by 49.9% (n = 500), for the depression vignette followed by 49.8% (n = 499) for social phobia, 39.5% (n = 396) for psychosis and 19.5% (n = 195) for the diabetes vignette. The response rate for exercise being a helpful intervention was 25% (n = 251) for the diabetes vignette, followed by 21% (n = 210) for social phobia, 18.7% (n = 187) for psychosis vignette and 18.4% (n = 184) for the depression vignette. While 70.2% (n = 704) responded that there would be benefit in seeing a doctor for the diabetes vignette, the response rates for psychosis was 48.5% (n = 486), and for both depression and social phobia it was 48.2% (n = 483). The responses for the persons in the vignettes becoming better with treatment was 81.4% (n = 816) for the diabetes, 79.5% (n = 797) for depression, 75.6% (n = 758) for psychosis and 63.4% (n = 636) for the social phobia vignette. A statistically significant association was found between the income level of the family and appropriate recognition as mental health problems, for all the 3 mental health related vignettes.

**Conclusions:**

The ability to recognise mental health problems, helpful interventions and outcomes in this population was comparable to those of adolescent populations in other countries, with some exceptions. The main differences were in relation to the identification and interventions in response to the psychosis and social phobia vignettes.

**Electronic supplementary material:**

The online version of this article (doi:10.1186/s13034-017-0176-1) contains supplementary material, which is available to authorized users.

## Background

Mental health literacy is a relatively new area of study, especially in developing countries. This concept has been defined as the ability to recognise specific disorders, knowing how to seek mental health information, knowledge of risk factors, causes of self-treatments, professional help available, and also, attitudes that promote recognition and appropriate help-seeking [1]. Studies indicate that in general, mental health literacy improves help seeking attitudes [2, 3].

There have been many large surveys on mental health literacy, worldwide. Most studies on adolescent populations have been conducted in developed countries [4–7]. Although there are a few such studies on adolescents in developing countries [8], most studies in these countries too have been focussed on adult populations [9, 10].

Current evidence shows that half of the lifetime diagnosable mental health disorders begin by the age of 14 years. This increases to three fourths by 24 years of age [11]. Hence, mental health literacy in adolescents has major implications for early identification and intervention of mental health issues. This early intervention can in turn, alter the developmental trajectory of mental illnesses and lead to improved outcomes [12]. Studies have shown that early help seeking prevents adverse social, educational and vocational outcomes in those with mental illness [13]. However, the number of adolescents who seek help for mental health related issues remain unsatisfactory. In the Australian National Survey of Mental Health and Wellbeing 2007 in young people aged between 16 and 24 years, only 32% of those with anxiety disorders, 49% of those with affective disorders and 11% of those with substance use disorders, had sought professional help during the previous 12 months [14].

In Sri Lanka, as per the most recent population census in 2012, 16.2% of the population is aged 10–19 years [15]. The prevalence rates of major mental illnesses in this age group in Sri Lanka, is not known. However, a national survey conducted in 2004 identified that 18.9% of 13–18 year olds had clinically relevant emotional and behavioural problems [16].

Although the level of mental health literacy among adolescents in Sri Lanka has not been described, reports are available on other youth groups. A recent study among undergraduates, found that only 7.4% were able to recognise and label a vignette describing the symptoms of depression [17]. Interestingly, more undergraduates indicated a preference for seeking informal help from friends and parents, rather than from psychiatrists and counsellors [17]. Studies among adolescents in developed countries such as Australia have provided important information for mental health initiatives aimed at improving health seeking and service delivery [5]. Serial surveys have found, poor recognition of disorders and negative beliefs about some standard psychiatric treatments, including medications. In contrast, there were positive views about self-help strategies, help from family and friends and psychological treatments such as counselling [5].

Sri Lanka has a relatively well developed western model of allopathic medical services. Mental health services using the western model are now available in all districts of the country. However, there are only a few dedicated Child and Adolescent Mental Health Services available currently. Furthermore, there are also practitioners of native healing methods mainly based on the Ayurvedic model. Meanwhile, especially in the case of emotional and behavioural issues, people also tend to practice Bodhi pooja. This is a traditional Buddhist cultural practice centred on a Bo tree (*Ficus religiosa*) which is a form of prayer. Some others seek solace in certain traditional ceremonies. A Thovil ceremony is a popular traditional healing ceremony with dancing and demonic masks, with the participants at times going into trance like states. These practices influence the knowledge and understanding of health, illness and help-seeking behaviour of the Sri Lankan population.

The objective of the current study was to describe aspects of mental health literacy in a school going sample of adolescents in Sri Lanka with regard to the (i) recognition of mental health problems (ii) helpful interventions, (iii) helpful referral options and (iv) outcomes. The association between socioeconomic variables and recognition of mental health problems was also examined.

## Methods

This was a descriptive cross sectional study. The setting was the Sri Jayawardenapura educational zone in the Colombo district of Sri Lanka. This is one of four educational zones in the Colombo District. The majority of students were expected to be between 13 and 16 years of age.

### Sampling size calculation

The sample size was calculated using the following formula [18].

1 $${\mathbf{n}} = \varvec{Z}_{{{\mathbf{1}} - \alpha /{\mathbf{2}}}}^{{\mathbf{2}}} \varvec{X}\,\frac{{\varvec{P}({\mathbf{1}} - \varvec{P})}}{{\varvec{d}^{{\mathbf{2}}} }} ,$$

 (where n is required sample size, $\varvec{Z}_{{{\mathbf{1}} - {\alpha \mathord{\left/ {\vphantom {\alpha {\mathbf{2}}}} \right. \kern-0pt} {\mathbf{2}}}}}^{{\mathbf{2}}}$ is Z value at 95% significant level = 2, P is expected prevalence, d is precision = 5%). For this calculation, the expected prevalence of good mental health literacy was assumed to be 50%, as used in previous studies where prevalence rates were unknown [10].

The number arrived at was 384. A further 10% was added to account for non-response or data recording errors. The number 422 thus obtained, was multiplied by 2 to counter the design effect. The final calculated minimum sample size was 844. This number was divided into 30 clusters, 15 clusters each from Grade 9 and Grade 10 (with a mean of 28 students in a cluster).

### Sampling method

**Fig. 1:**
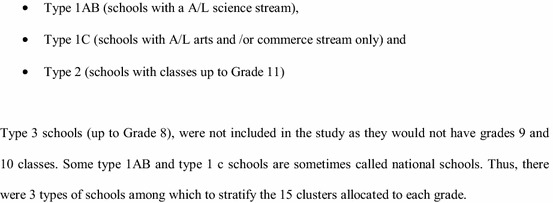
Explanation of the categorisation of schools

Multi-stage, cluster-sampling was used in identifying the study sample and to include a representative cross section of the school children. This was necessary because the geographical area included several categories of schools with wide ranging classroom numbers, resource availability and courses of study (Fig. 1).

A classroom with students between the minimum number [15] and the maximum number (40) was considered a cluster (mean 28). Allocation of clusters, to each category of school was done in proportion to the number of classrooms fulfilling the criteria for a cluster, in each category. This data were obtained from the relevant educational authorities. Thereafter cluster allocation was done randomly within the different categories of schools. While there were in total 96 schools in the education zone, after final cluster selection 46 schools were included in the study (type 1AB schools-14, type 1C-8 schools and type 2 schools 24).

### Method of data collection

Students in the selected classrooms were given the questionnaire after obtaining prior informed consent from the school principal and class teachers. Consent was also obtained from the Director in charge of this education zone.

Prior, informed, written, consent was also obtained from the parents of the students in the selected classes. Information sheets about the study were also given along with the consent forms. Only those who returned the consent forms were included in the study. The assent of the participants themselves was asked for, before the questionnaire was given. Filling in the forms, on average, took around 20 min per questionnaire. The data collection was done within a 12 week period.

### Assessment of mental health literacy

The adolescents included in the study responded to a questionnaire based on 4 clinical vignettes. The design of the questionnaire modelled that of the Australian National Survey on mental health literacy 2011 [5]. The current study used several vignettes, and questions based on the vignettes used in this study. The format of this survey had evolved and developed after several such surveys in the past years. The case vignettes used in our study, were on depression with suicidal ideation, social phobia, (Additional file 1) psychosis and diabetes mellitus. The vignette on diabetes mellitus (a physical health condition) was included for comparison. The questionnaire was initially written in English and translated to Sinhala and Tamil (the 2 main languages spoken in Sri Lanka). The questionnaire was tested for face and content validity using the Delphi method. Those participating in this process included, child and adolescent psychiatrists, psychiatrists and medical officers with experience in adolescent psychiatry (6 participants in all). The Delphi process was stopped only after all in the panel agreed on the content, structure, representativeness of the vignettes, cultural appropriateness of the questions and the suitability and appropriateness of the responses to the questions, after 5 rounds of discussions.

The questionnaire was first administered to a representative sample of students in another similar education zone. This pilot study was conducted to establish (i) acceptability of the (Additional file 2) questions, (ii) comprehension of the vignettes and questions, (iii) ease of administration, (iv) to refine the method of administration, (v) to assess the completeness of returned questionnaires, (vi) participation rates and (vii) to calculate the average time needed for completion. Test retest reliability was assessed by administering the questionnaire to a few of the pilot study participants a week later.

The final questionnaire consisted of close ended questions based on the vignettes. The questions required the respondents to give their opinion on, (i) whether the vignette depicted a mental health related problem, spiritual, physical related or other problem, (ii) what interventions could be helpful, (iii) what kinds of referrals would be helpful and (iv) possible outcomes for each vignette. Respondents were allowed multiple answers, as it was likely that this would be the situation in reality. The family income and the educational level of each parent, was included in the analysis as these socio-economic indicators were more likely to have an impact on mental health literacy.

The data was analysed for frequency distribution, cross tabulation and Chi square testing, using the Statistical Package for Social Sciences (SPSS) version 16 software package. The significance level for this study was set at 0.05.

## Results

Although 1500 participants aged 13–16 years were initially approached for consent, the final data number was 1002 or 67% of the initial number of participants approached. The rest of the consent forms were either not returned or the adolescents had not responded to the questionnaire. Although the option of a Tamil questionnaire was provided only the Sinhala questionnaire had been used.

The mean age was 14 years (SD ± 0.94). Of the participants 590 (58%) were male.

Table 1 describes the responses regarding helpful intervention options, helpful referral, recognition of problems and outcomes.

**Table 1:** Responses for helpful intervention options, referral options, recognition of problems and outcomes

	Person in the vignette can be helped by	Number (%) depression	Number (%) social phobia	Number (%) in psychosis	Number (%) in diabetes
Helpful intervention options	Talking to him	500 (49.9)	499 (49.8)	396 (39.5)	195 (19.5)
	Physical exercise	184 (18.4)	210 (21.0)	187 (18.7)	251 (25.0)
	Introducing him to a new hobby	346 (34.6)	282 (28.1)	219 (21.9)	145 (14.5)
	Referral to a health service	465 (46.5)	417 (41.6)	528 (52.7)	698 (69.6)
	By any other method	128 (12.8)	35 (3.5)	214 (21.4)	110 (11.0)
	Did not answer	12 (1.2)	20 (2.0)	24 (2.4)	33 (3.3)
Referral options	Bodhi pooja	299 (29.9)	215 (21.5)	318 (31.7)	141 (14.1)
	Thovil ceremony	86 (8.6)	124 (12.4)	158 (15.8)	107 (10.7)
	A doctor in the government sector	483 (48.2)	483 (48.2)	486 (48.5)	704 (70.4)
	A native doctor	229 (29.9)	185 (18.5)	206 (20.6)	214 (21.4)
	Another service	153 (15.3)	314 (31.3)	179 (17.9)	112 (11.2)
	Did not answer	23 (2.3)	23 (2.3)	26 (2.6)	35 (3.5)
Recognition as a physical/mental/social/spiritual/other problem	A physical problem	275 (27.2)	265 (26.5)	225 (22.5)	586 (58.48)
	A mental problem	824 (82.2)	623 (62.3)	689 (68.7)	389 (38.82)
	A social problem	191 (19.1)	269 (26.9)	169 (16.9)	98 (9.78)
	A spiritual problem	35 (3.5)	36 (3.6)	223 (22.3)	49 (4.89)
	A behavioural problem	83 (8.3)	164 (16.4)	168 (16.8)	87 (8.68)
	Another problem	35 (3.5)	66 (6.6)	82 (8.2)	138 (13.77)
	Did not answer	23 (2.3)	25 (2.5)	27 (2.7)	37 (3.69)
Responses regarding outcomes	Not be able to get back his usual lifestyle	19 (1.9)	27 (2.69)	42 (4.2)	40 (4.0)
	Will recover on his own	58 (5.8)	337 (33.6)	202 (20.)	130 (13.)
	Will become better with treatment	797 (79.5)	636 (63.47)	758 (75.6)	816 (81.4)
	Did not answer	21 (2.1)	60 (5.98)	67 (6.7)	54 (5.4)

A Bodhi pooja is a traditional Buddhist cultural practice centred on a Bo tree (*Ficus religiosa*), this is a form of pooja (idolatry or prayer). A thovil ceremony is a traditional healing ceremony with dancing and sometimes the involvement of demonic masks, with the participants going into trance like states at times

The response rates for recognition as a mental health problem was 82.2% (n = 824) for the vignette depicting depression, 68.7% (n = 689) for the psychosis vignette and 62.1% (n = 623) for the social phobia vignette. Meanwhile 58.48% (n = 586) responded to the diabetes vignette as a physical problem. Of the four vignettes, social phobia had the highest response rate as a social problem at 26.9% (n = 269). The psychosis vignette had the highest rate of response at 22.3% (n = 223) as a spiritual problem.

In the current study, the highest response rate for the helpfulness of referral to a health service was for the diabetes vignette at 69.6% (n = 698), followed by psychosis 52.7% (n = 528).

“Talking to the person”, was responded to as helpful by 49.9% (n = 500), in the depression vignette followed by 49.8% (n = 499) in the social phobia vignette, 39.5% (n = 396) in the psychosis vignette and 19.5% (n = 195) in the diabetes vignette. The response rates for both depression and social phobia were significantly different (P < 0.001) from that of psychosis.

Exercise was responded to as being helpful by 25% (n = 251) in the diabetes vignette, followed by 21% (n = 210) in the social phobia, 18.7% (n = 187) in the psychosis and 18.4% (n = 184) in the depression vignettes. The response for the diabetes vignette was significantly different (P < 0.005), from all three mental health related vignettes.

While 70.2% (n = 704) responded that there would be benefit in seeing a doctor for the diabetes vignette, the response rates for psychosis was 48.5% (n = 486), and for both depression and social phobia it was 48.2% (n = 483). The response rate for diabetes was significantly different from (P < 0.0001) from the other vignettes.

Meanwhile, 31.7% (n = 318) responded to the option “Bodhi pooja” as a helpful referral for psychosis. The corresponding figures for depression and social phobia were 29.7% (n = 299) and 21.5% (n = 215). The response rate for psychosis while not significantly different from depression, was significantly different from both diabetes and social phobia (P < 0.001).

The responses for the persons in the vignettes becoming better with treatment was 81.4% (n = 816) for diabetes, 79.5% (n = 797) for depression, 75.6% (n = 758) for psychosis and 63.47% (n = 636) social phobia. With regards to social phobia, 33.6% (n = 337) also responded that the person would become better on their own.

Table 2 describes the association between several socio-economic variables, namely the fathers’ education level, mothers’ education level, and monthly family income with the ability to recognise mental health issues. The fathers’ education level was significantly associated with better recognition of mental health problems in the depression and psychosis vignettes (P values 0.001 and 0.003). However, a higher monthly family income was the only variable that was significantly associated with appropriate identification of a mental health issue across all 3 mental health related vignettes (depression—Chi square 8.271, P value 0.004, social phobia—Chi square 9.17, P value 0.002, psychosis—Chi square 17.29, P value < 0.001).

**Table 2:** Cross tabulation—fathers education level, mothers education level, monthly family income level and associations with the ability to recognise mental health problems

	Depression		Social phobia		Psychosis		Diabetes	
	Yes %	Not identified %	Yes %	Not identified %	Yes %	Not identified %	Yes %	Not identified %
Income								
<Rs 20,000	19.4	21.3	20.1	20.5	34.3	6.4	33.0	7.6
Rs 20,000 and above	24.5	34.3	26.9	32.5	47.0	12.4	45.2	14.1
Total	43.9	55.6	47.0	53.0	81.3	18.8	78.2	21.7
Chi square value	8.271		9.17		17.29		2.65	
df	1		1		1		1	
P value	0.004		0.002		<0.001		0.103	
Fathers highest education level								
Passed GCE O/L’s or below	21.2	26.4	22.7	24.9	38.5	9.1	38.0	9.6
Above O/L’s to University	22.6	29.8	23.7	28.6	43.1	9.3	40.6	11.8
Total	43.8	56.2	46.4	53.5	81.6	18.4	78.6	21.4
Chi square value	11.8		0.695		8.812		2.7	
df	1		1		1		1	
P value	<0.001		0.40		0.003		0.1	
Mothers highest education level								
Passed GCE O/L’s or below	20.4	27.6	20.8	27.2	39.2	8.8	37.3	10.7
Above O/L’s to University	24.0	28.0	25.8	26.2	42.5	9.5	41.2	10.8
Total	44.4	55.6	46.6	53.4	81.7	18.3	78.5	21.5
Chi square value	3.29		3.29		0.89		11.214	
df	1		1		1		1	
P value	0.07		0.07		0.345		<0.001	
Overall	46.4	53.6	48.3	51.7	72.9	27.1	79.6	20.4

One Sri Lankan rupee is equivalent to around 0.007 U.S D

*GCE O/L’s* Government Certificate Examination Ordinary level. This is a standardised exam held after 10 years of schooling, *GCE A/L’s* Government certificate Examination Advanced level. This standardised examination is held after a further 2 ½ years and is the entrance exam for university education, *df* degrees of freedom

## Discussion

### Ability to recognise mental health problems

In this study, most respondents could recognise the depression, psychosis and social phobia vignettes as mental health problems. This was more than the number of respondents who recognised diabetes as a physical problem (Table 1).

However, this study assessed the ability of the participants to recognise a problem affecting the mental wellbeing, rather than an ability to give a diagnostic label of depression, social phobia or psychosis.

In Sri Lanka, there are no popular lay term for depression and other mental health disorders in local languages. Terms such as “vishadaya” for depression are unfamiliar to the general population [19–21]. This difficulty may explain why in a previous study, the recognition of depression among undergraduates in Sri Lanka was as low as 17.4% [17]. This rate fell further when medical undergraduates were excluded from the analysis.

In international studies, the recognition of disorders in vignettes varies widely, with depression, being the most easily recognised disorder [5]. Psychosis and social phobia had lower rates of recognition.

In this study too, the symptom complex of depression was the most easily recognised, and that of social phobia the least recognise as a mental health problem. It is possible that adolescents in the study population found it difficult to differentiate between normal social anxiety and social phobia.

Cultural influences may explain the high rate of responses for the psychosis vignette as being a spiritual problem. Belief in the supernatural and dissociative disorders, presenting with psychosis like hallucinations and delusions are well recognised in the Indian sub-continent [22].

However, in comparison to other such questionnaires our questionnaire did not include open ended questions and the response choices were limited. Having open ended questions and a broader response choice may have yielded more information. While our study was on the recognition of a mental health problem, other studies required the identification of specific mental health disorders (such as depression) as well. This may have an influence in comparing our study with other such studies.

### Helpful interventions

In the current study, the highest response rate for the helpfulness of referral to a health service was for the diabetes vignette (69.6%), followed by psychosis (52.7%), depression (46.4%) and social phobia (41.61%) vignettes.

A relatively high response rate, across all the mental health related vignettes was seen for “talking to the person”, as a helpful intervention. This was highest for the social phobia vignette (50%) followed by depression (49.8%), psychosis (39.5%) and diabetes (19.5%) (Table 1). In comparison, in the 2011 National Mental Health Literacy Survey in Australia (youth component), the rates for “helping by talking to the person” were 52.2% for depression, 48.9% for psychosis and 46.4% for social phobia [5]. The differences in rates in the current study may again be due to cultural influences. It may be perceived that talking to a person with anxiety is more appropriate and acceptable, than talking to a person exhibiting delusions and hallucinations.

Interestingly although depression was better identified as a mental health, problem, those with psychosis were more likely to be referred to a health service. This may be an indication that depression is considered a less severe condition, with less urgency for referral to health services.

Surprisingly the number, who had responded that exercise would be beneficial in depression, was less than that for all other vignettes. In contrast, in a study among university undergraduates in Sri Lanka, 85.6% responded that physical exercise is a helpful intervention in relation to the given depression vignette [17]. It is possible that the link between mental well-being and physical activity was not understood by adolescents as in the undergraduate age group.

### Helpful referral options

The response rate for referral to a medical doctor in the physical health vignette (diabetes) was significantly higher than for the mental health vignettes (Table 1). This indicates that respondents were likely to be more comfortable in seeking the support of a doctor for physical symptoms, as opposed to behavioural, emotional and cognitive symptoms, which are the common manifestations of mental ill health.

While the response rate for referral to a medical doctor for the psychosis vignette was 48.5%, the response rate for the benefit of a Bodhi pooja was 31.7%. A Bodhi pooja is a religious ritual centred on a Bo tree (*Ficus religiosa*). This ritual is derived from Buddhist cultural practices. This finding is consistent with the relatively higher culturally influenced response rates for a spiritual causation for psychosis. Similarly, a study in an adult population in India highlighted how cultural factors effect referral and help seeking options [23]. In this study 74% responded that mental illness is related to evil spirits or black magic and possibly due to sins in one’s past life. The same percentage also responded that going to a traditional healer would improve the condition [23].

### Outcomes

A majority responded that the persons in all the vignettes would become better with treatment (Table 1). In the Australian National Mental Health Literacy Survey 2011; respondents in the general community survey were also more likely to believe in full recovery in relation to similar vignettes with problems re-occurring for those who received appropriate treatment for mental disorders [5].

Interestingly 33.63% had responded that the person in the social phobia vignette would recover on their own, but the corresponding response was 5.8% for depression. Even so, 48.2% considered a doctor in the government sector as a referral option in the social phobia vignette, which was similar to that for the depression vignette.

Around 20% also responded that the person depicted in the psychosis vignette would recover on their own. The reason for this is unclear, but it is possible that these adolescents are more familiar with transient psychotic like states, rather than chronic psychotic disorders such as schizophrenia.

### Socio economic variables and mental health literacy

A higher socio-economic level was significantly associated with better recognition of mental health problems and appropriate referral (Table 2). Meanwhile when parental education levels were considered, the fathers’ education level was significantly associated with better recognition of mental health problems in only the depression and psychosis vignettes. The mothers education level was not significantly associated with recognition of any of the mental health related issues.

These finding contrasts with the finding of studies in other parts of the world, where increasing parental education levels have been significantly associated with higher mental health literacy levels [24]. The differences in the finding of the current study may be related to the differences in the parental education levels between the sample populations. In the current sample only 52.4% of fathers and 52% of mothers were reported to have continued education beyond the G.C.E O/L exam.

These findings however, point to the need for mental health initiatives in the future to specifically target populations in the lower income range.

## Limitations of the current study

This study was limited to a school going population of adolescents. It is possible that the responses would have been different among school dropouts. Studies have also found that in some countries, mental health problems alone can be a major contributor towards dropping out of school [25].

A difficulty in generalising the findings of the current study, to other geographical areas of Sri Lanka with different socio-economic conditions is another issue.

Participant responses to case vignettes may not necessarily indicate reality [26]. Courtesy bias, as well as recall bias, both affects the response rates. The study instrument was a self-administered questionnaire based on vignettes rather than a rating scale. This meant that mental health literacy levels could not be scored. However; such vignette based questionnaires have been used in similar studies across the world providing valuable results [5, 17].

In comparison to other such questionnaires our questionnaire did not include open ended questions and the response choices were limited. Having open ended questions and a broader response choice may have yielded more information.

## Conclusions

The ability to recognise mental health problems, helpful interventions and outcomes in this population was generally comparable to those of adolescent populations in international studies with some exceptions. The main differences were in relation to the identification and interventions in response to the psychosis and social phobia vignettes. Why mental health issues were better recognised but the physical health issue was more likely to be referred, is an important area to be explored further. This is likely to have major implications for mental health initiatives for adolescents in the future, including methods for promoting mental health literacy.

## Additional files

**Additional file 1.** The Case vignettes.

**Additional file 2.** The question format.

## Abbreviations

G.C.E: General Certificate in Education
O/L: ordinary/level
A/L: advance/level
SPSS: Statistical Package for Social Sciences
