# Supplementary material for: Mental health literacy in adolescents: ability to recognise problems, helpful interventions and outcomes
Source: Child Adolesc Psychiatry Ment Health. 2017 Aug 15;11:38. doi: 10.1186/s13034-017-0176-1 (PMC5557470; doi:10.1186/s13034-017-0176-1)
Supplement: Supplementary file 1 — Additional file 1. The Case vignettes. [file 13034_2017_176_MOESM1_ESM.docx]

**Case vignettes**

**1. Depression with suicidal ideation**

Manju is an 18 years old Student. He has not been going to School for the past one month complaining of fatigue and low mood. He finds it difficult to fall asleep, complaints of headaches, difficulty concentrating and irritability. He feels he has no escape from his problems and feels worthless. He has contemplated ending his life at times.

**2. Social Phobia**

Geethani is an 18 year old student. She has extreme fear of talking in the presence of others. On these occasions she feels faintish, is sweaty and shivers. She avoids these situations and feels extremely fearful even when thinking of such situation. With these symptoms she has been having many difficulties in continuing her school work.

**3. Psychosis**

Nishantha is an 18 year old student since about a month he has been refusing to go to school he prefers to stay at home with doors and windows shut.

His parents complain that he sleeps poorly at night, laughs and talks on his own. He claims he is responding to voices he hears.

He does not maintain self-care and claims that he remains at home as his neighbors trouble him. He believes he has no illness.

**4. Diabetes Mellitus**

26 year old Saman has since of late been feeling extremely tired, and has lost his appetite and also lost weight markedly. He complains of increased frequency in passing urine and has to even wake up at night to relieve himself.
