# Supplementary material for: Mental health literacy in adolescents: ability to recognise problems, helpful interventions and outcomes
Source: Child Adolesc Psychiatry Ment Health. 2017 Aug 15;11:38. doi: 10.1186/s13034-017-0176-1 (PMC5557470; doi:10.1186/s13034-017-0176-1)
Supplement: Supplementary file 2 — Additional file 2. The question format. [file 13034_2017_176_MOESM2_ESM.docx]

The following questions were given after all 4 vignettes with the instructions

*There will be four scenarios of four people described below. Please answer about your ideas*

*and decisions regarding the questions asked about them.(you can choose more than one*

*answer for each question)*

According to you

- 1. The above mentioned person can be helped by

1. Talking to him/her
2. Physical exercise
3. Introducing him/her to a new hobby
4. Referring him/her to a health service
5. By any other method
   1. It will be appropriate to direct him/her to the following

1. A Bodhi Pooja

1. Thovil ceremony
2. A Doctor in the government sector

1. A Native Doctor

1. Another service

3) His/ Her problem is a

1. a spiritual problem

1. a physical problem

1. a mental problem

1. a social problem

1. a behavioral problem

1. Other

1. According to you he/she will

- - 1. Not be able to get back his/her usual lifestyle
    2. Will recover on his/her own

- - 1. Will become better with medication
